# Post-weaning diarrhea and use of feedstuffs in pigs

**DOI:** 10.1093/af/vfac079

**Published:** 2022-12-14

**Authors:** Liuqin He, Xiaoya Zhao, Jianzhong Li, Chengbo Yang

**Affiliations:** Hunan Provincial Key Laboratory of Animal Intestinal Function and Regulation, Hunan International Joint Laboratory of Animal Intestinal Ecology and Health, College of Life Sciences, Hunan Normal University, Changsha 410081, China; College of Animal Science, South China Agricultural University, Tianhe District, Guangzhou 510642, China; Hunan Provincial Key Laboratory of Animal Intestinal Function and Regulation, Hunan International Joint Laboratory of Animal Intestinal Ecology and Health, College of Life Sciences, Hunan Normal University, Changsha 410081, China; Department of Animal Science, University of Manitoba, Winnipeg, MB R3T 2N2, Canada

**Keywords:** acid-binding capacity, essential oils, post-weaning diarrhea, sodium metabisulfite, vomitoxin

**Implications**

- The role of living microbes on swine health is undeniable. However, antimicrobial properties should not be solely based on selecting essential oils in swine production. Instead of higher doses required for their killing pathogens properties, using lower doses of essential oils could prevent inflammation and “leaky gut” induced by lipopolysaccharide.
- It is critical to look at the total blend of raw materials available for feed formulation, their inclusion levels, costs, feed preference as well as the targeted feed acid-binding capacity value and then make the final decision based on science and field experience.
- Minimizing mycotoxin contamination in feeds is an important component to control post-weaning diarrhea. Chemical approaches, such, as the use of sodium metabisulfite and biological approaches, such as the use of microorganisms for detoxification, have shown promise in reducing vomitoxin.

## Introduction

Weaned piglets are highly susceptible to many stressors including bacterial pathogens, oxidative stress, and inflammation, which predisposes pigs to post-weaning diarrhea, eventually leading to reduced growth performance, high mortality and morbidity rates, and compromised animal welfare (Yang et al., 2015a, 2015b; Hassan et al., 2018). Since post-weaning diarrhea commonly results from the proliferation of pathogenic *Escherichia coli*, antibiotic growth promoters have been widely used in piglet diets, especially in nursery diets, to control incidences of diarrhea during the transition. Total consumption of antimicrobials in animal food production worldwide was estimated at 63,151 tons in 2010, with an increasing trend; the annual consumption of antimicrobials per kilogram body weight was 148 mg/kg for pigs (Van Boeckel et al., 2015). This practice may lead to the spread of antimicrobial-resistant bacterial pathogens in pigs and humans, challenging the sustainability of the pork industry (Yang et al., 2015a, 2015b). With environmental, health, and safety concerns, the public demands antibiotic-free pork (e.g., raised without antibiotics). However, the withdrawal of antibiotics from feeds can result in several challenges including compromised gut health and increased gut diseases. So far, we do not have a single “magic bullet” that can replace in-feed antibiotics. Although different types of alternatives to antibiotics (e.g., essential oils and probiotics) have been widely recognized as promising alternatives to antibiotics in feeds, an integrated approach to control post-weaning diarrhea should be taken, including supplementation of antibiotic alternatives, and measures related to nutrition, biosecurity, and management. In this review article, we reviewed the use of essential oils as antibiotic alternatives, the use of ingredients to lower dietary acid-binding capacity (ABC), and the use of innovative chemical and biological approaches to detoxify vomitoxin, which may be considered important parts of an integrated approach to control post-weaning diarrhea in piglets.

## Using Essential Oils as Antibiotic Alternatives

**Figure 1.**
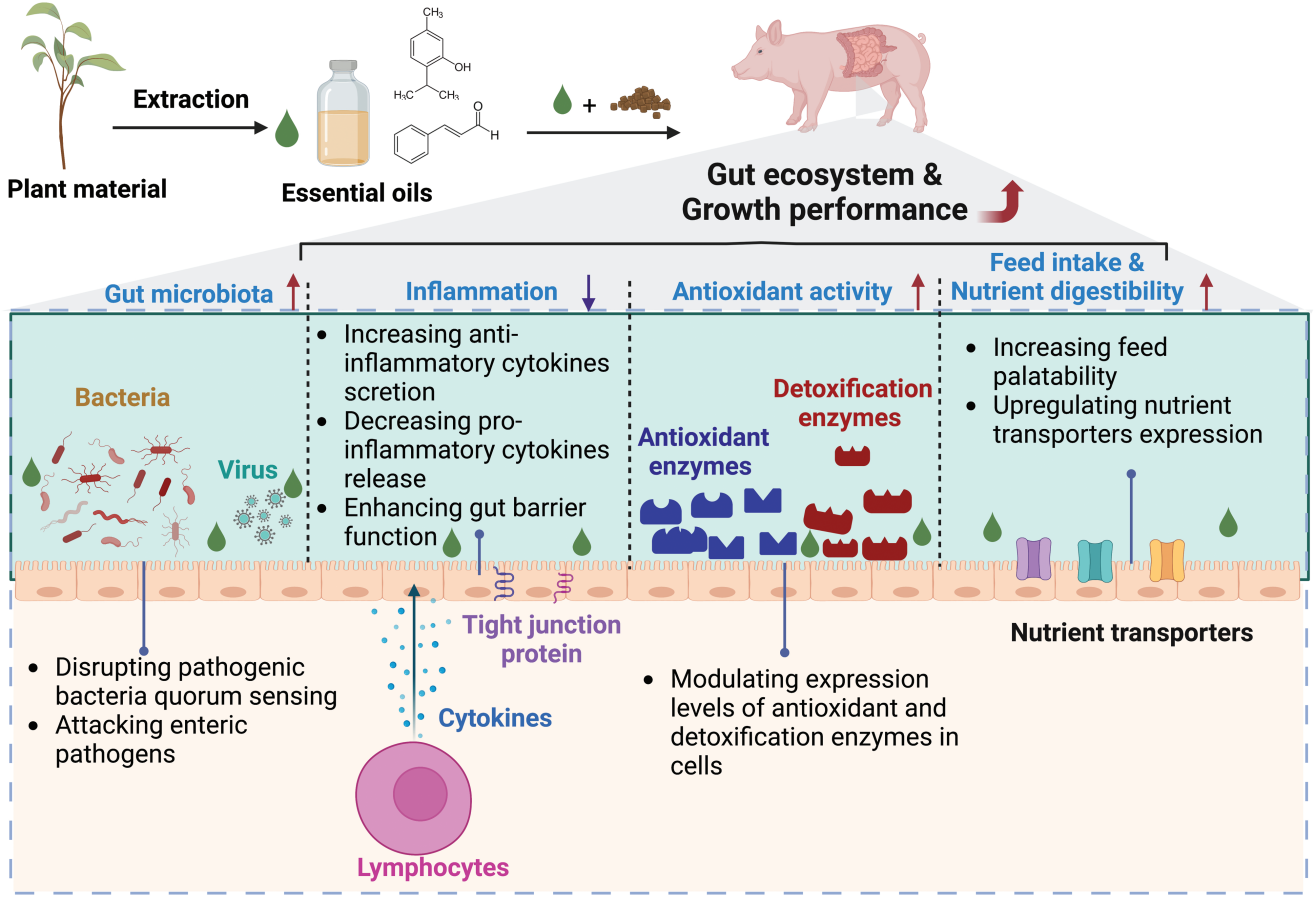
Schematic diagram illustrating the four different potential mechanisms by which essential oils improve the gut ecosystem and growth performance of piglets.

Essential oils have antioxidative, anti-inflammatory, and antimicrobial properties. Some essential oils (e.g., thymol, eugenol, and cinnamaldehyde) have been widely used to replace antibiotics in swine production mainly because of these essential oils’ antimicrobial properties (Yang et al., 2015a, 2015b; Hassan et al., 2018; Omonijo et al., 2018a). The adverse effects of pathogenic microbes on swine health are undeniable. However, antimicrobial properties should not be solely based on selecting alternatives to antibiotics in swine production (Gresse et al., 2017; Fouhse et al., 2016; Figure 1). Lipopolysaccharides, also called endotoxins, are cell wall components of Gram-negative bacteria (e.g., *Salmonella* and *Escherichia*) that are present everywhere in the environment including in the intestine, ground, air, and water, and have received much attention due to their ability to stimulate a low-grade inflammation in pigs (Huang et al., 2016; Nordgreen et al., 2018). One of the negative consequences of inflammation at the intestine level is increased intestinal permeability, or “leaky gut,” associated with impaired nutrient absorption and increases in diarrhea incidence (Gresse et al., 2017). Interestingly, in addition to antimicrobial properties, thymol (50 µM, 7.5 mg/kg), eugenol (100 µM, 16.4 mg/kg), and citral (10 to 20 µM, 1.52 to 3.04 mg/kg) can reduce inflammation associated with lipopolysaccharide or peptidoglycan in porcine intestinal epithelial cells (Omonijo et al., 2019; Hui et al., 2020; Li et al., 2022a, 2022b) and cinnamaldehyde (25 µM, 3.3 mg/kg) can improve intestinal mucosal barrier function (Sun et al., 2017). These levels are much lower than the minimum inhibitory concentration of thymol and eugenol against major pathogens (Yang et al., 2015a, 2015b; Omonijo et al., 2018a). Moreover, the minimum inhibitory concentration of most essential oils is much higher than the acceptable levels in the animal industry in terms of cost-effectiveness and feed palatability (Omonijo et al., 2018a). Since the industry’s acceptance of essential oils also depends on inclusion cost, those results are encouraging producers to use lower doses of essential oils to prevent inflammation and “leaky gut” induced by lipopolysaccharide instead of using higher doses required for their killing pathogens properties. Moreover, using higher doses has drawbacks on optimal feed intake and cost.

The combined use of essential oils and other additives should lead to higher advantages. For two reasons, a combination of other alternatives with essential oils holds the most promise as a substitute for antibiotics in pig feeds. First, no single antibiotic alternative has been reported to be able to replace antibiotics completely. Second, a combination of products can have a synergistic effect that will reduce the effective dosages required to combat pathogens (e.g., organic acids and essential oils). For example, essential oil can change the structure and functions of bacterial cell membranes. This results in membrane swelling and thus increased membrane permeability, leading the bacteria toward an increased susceptibility to organic acids. Moreover, the hydrophobicity (being water-repellent) of essential oil is increased at low pH. Combining essential oils with organic acids will enable the essential oil to pass through the lipids of the bacterial cell membrane more easily.

Essential oils exhibit great potential to prevent post-weaning diarrhea. However, their direct inclusion in pig diets has compromised efficacy because of such factors as low stability, poor palatability, and low availability in the lower gut. Therefore, an effective and practical delivery method is very important for the use of essential oils in swine production. It is documented that the formulation of microparticles could effectively deliver thymol and lauric acid to the pig intestinal tract. Lauric acid not only acts as a carrier for thymol but also has synergistic antibacterial effects with thymol (Omonijo et al., 2018b). The stability of thymol in commercial lipid matrix microparticles (encapsulated essential oils and organic acids) was investigated during feed pelleting and feed storage and determined the intestinal release of thymol (Choi et al., 2020a). The thymol concentration was not significantly different in the mash and pelleted feeds, suggesting that the pelleting of feed did not affect total thymol in those lipid matrix microparticles. Encapsulated thymol was also stable in simulated pig gastric fluid (26.0% thymol released). The rest of the thymol was progressively released in the simulated intestinal fluids until completion, which was achieved within 24 h. In a pig experiment, 15.5% of thymol was released in the stomach, and 41.9% of thymol was delivered in the mid-jejunum section, demonstrating a slow release, and 2.2% of thymol was recovered in feces (Choi et al., 2020a). The lipid matrix microparticles maintained the stability of thymol during the feed pelleting process and storage and allowed a slow and progressive intestinal release of thymol in weaned pigs (Choi et al., 2020a). Subsequent studies were conducted to investigate further the effects of these commercial lipid matrix microparticles on growth performance, immune system, gut barrier function, nutrient digestion, and absorption in disease-challenged weaned piglets and demonstrated that the supplementation of those lipid matrix microparticles showed anti-diarrhea effects in disease-challenged weaned piglets (Choi et al., 2020b; Xu et al., 2020). Therefore, microencapsulated essential oil and organic acid combination can be a useful method to control post-weaning diarrhea in swine production.

## Using Probiotics to Improve Gut Health

Direct-fed probiotics refer to live microorganisms supplied to the host with adequate amounts benefiting the host (Pluske, 2013). The benefits of providing direct-fed probiotics into swine diets are categorized into several aspects:1) benefiting gut health by modifying the composition of enteric microflora (Sartor, 2004); 2) promoting immunity (Yan and Polk, 2011), 3) increasing efficiency of nutrient digestion and utilization (Yadav and Jha, 2019) and 4) enhancing gut function and improving growth performance (Vohra et al., 2016). Direct-fed probiotics contain three main categories: *Bacillus*, lactic acid-producing bacteria, and yeast (Kerr et al., 2013). *Bacillus* is a potent producer of extracellular fiber-degrading enzymes, which can increase nutrient digestibility and utilization (Kiarie and Mills, 2019). In addition, *Bacillus* synthesizes enzymes that degrade feed and produces short-chain fatty acids through fermentation (Merchant et al., 2011). Those short-chain fatty acids are considered a useful energy source utilized by pigs to develop the large intestine (den Besten et al., 2013). Recent studies have identified the protective effect of *Bacillus* strains on intestinal cells challenged with enteric pathogenic bacteria, since they found the pre-treatment of *Bacillus* could upregulate tight junction protein expression and decrease quorum sensing (Chen et al., 2021; Li et al., 2022a, 2022b). For weaned piglets, including lactic acid-producing bacteria in their dies could relieve weaning stress, reduce diarrhea, and enhance growth performance (Yang et al., 2015a, 2015b). Lactic acid-producing bacteria are the primary bacteria in the nursing pig gut, and the lactic acid produced by lactic acid bacteria fermentation can inhibit the growth of intestinal pathogenetic bacteria and helps to aid immunity (Guevarra et al., 2019). That makes the supplementation of lactic acid-producing bacteria beneficial for the weaned piglets (Guevarra et al., 2019). The common forms of yeast supplied to pig diets include whole live yeast cells, heat-treated yeast cells, ground yeast cells, purified yeast cell cultures, and yeast extracts. Yeast supplementation has been reported to boost intestinal development by providing fermentation by-products such as short chain fatty acids to pigs and reducing post-weaning scour by supplying weaned piglets with beneficial nutrients such as specific sugars and nucleotides (Broadway et al., 2015). Accordingly, introducing probiotics in the creep feed is increasingly being explored (Barba-Vidal et al., 2018). However, the results of these studies are not consistent and further studies are still needed to interpret the mechanisms of action of probiotics and their interaction in various gut health situations.

## Using Ingredients to Lower Dietary Acid-binding Capacity

Due to the decreased capacity of gastric acid secretion at weaning, weaned piglets have a higher pH value in the stomach than sow-reared piglets (Heo et al., 2013). Maintaining a lower gastric pH value is pivotal for the gut health of weaned piglets because this can positively affect the nutrition digestion and pathogenic bacteria inhibition. In contrast, the elevated gastric pH level makes weaned piglets more susceptible to enteric infections (Heo et al., 2013). Hence, not only the amino acid profile or the energy content of the diet but also other nutrients and key parameters should be considered (e.g., dietary ABC, Table 1). It refers to the ration’s resistance to a low pH in the pig’s stomach, is highly related to raw materials used in the feed and has a great impact on the pH of the stomach and feed digestibility. A high ABC can lead to lower digestibility of dry matter and crude protein and, therefore, adversely affect the growth performance of piglets. Moreover, a high ABC can increase the release of amine and ammonia that are toxic and could lead to diarrhea. The ABC value of feed ingredients and complete feeds can be calculated as the amount of acid in milliequivalents (meq) required to lower the pH of 1 kg of a sample to pH 4 and pH 3 based on the measurement value from a 0.5 g sample (Lawlor et al. 2005) and it can be used a nutrient constraint in feed formulation to select suitable ingredients. Therefore, lowering the ABC of the diets for new weanlings with the addition of feedstuffs with a low ABC in piglet feed formulations can be a good strategy for controlling diarrhea in weaned piglets (Huting et al., 2021).

**Table 1.** Acid-binding capacity for different ingredients (Scholten et al., 2001; Lawlor et al., 2005; Karvelis, 2014; Hajati, 2018)

Ingredient	ABC-3^*a*^	ABC-4^*b*^	Unit
Milk			
Acid casein	200	0	meq/kg
Sows milk	650	481	meq/kg
Whey powder	714 − 1,000	434	meq/kg
cheese whey	60.0 − 48.8		meq/kg
Milk replacer	892	579	meq/kg
Skim milk	1,105	756	meq/kg
Rennet casein	1,929	1,423	meq/kg
Cereals			
Wheat Soft	250		meq/kg
Wheat bran	500		meq/kg
Wheat	180 − 40	108	meq/kg
liquid wheat starch	77.0 − 74.5		meq/kg
Maize	200 − 254	111	meq/kg
Barley	225 − 266	113	meq/kg
Maize starch	202	91	meq/kg
Corn	135 − 172		meq/kg
Corn distillers	438	96	meq/kg
Oat flakes	180	72	meq/kg
Root and pulp products			
Sugar	98	23	meq/kg
Cassava	393	167	meq/kg
Beet pulp	480	191	meq/kg
Molasses	790	399	meq/kg
Citrus pulp	873	373	meq/kg
Mashed potato steam peel	64.2 − 79.5		meq/kg
Vegetable protein			
Sunflower meal	852	482	meq/kg
Rapeseed meal	945	498	meq/kg
Soybean meal	1,068	642	meq/kg
Soybean meal 42%	980 − 1,240		meq/kg
Soybean meal 44%	1,100		meq/kg
Soybean meal 48%	1,025 − 1,035		meq/kg
Palm kernel	485	250	meq/kg
Peas	515	278	meq/kg
Maize gluten	571	114	meq/kg
Beans	473	275	meq/kg
Meat and fishmeal			
Blood plasma	1,150 − 1,350		meq/kg
Meat and bone meal	920	595	meq/kg
Fishmeal	1,122.5 − 2,100	738	meq/kg
Fishmeal 70/72%	1,800 − 2,200		meq/kg
Fishmeal Peru origin	1,800 − 2,000		meq/kg
Fat			
Fat	137	16	meq/kg
Vegetable fat	200		meq/kg
Choline chloride	100 − 226	101	meq/kg
Betaine	600		meq/kg
Dextrose	140 − 200		meq/kg
Microbial protein			
Yeast	130	150	meq/kg
Amino acids			
Lysine	600 − 695	123	meq/kg
Tryptophan	1,024	179	meq/kg
Methionine	1,000 − 1,219	192	meq/kg
Threonine	1,100 − 1,386	218	meq/kg
Minerals			
Limestone	18,500 − 22,000		meq/kg
Ferrous sulphate	93	−655	meq/kg
Salt	162	83	meq/kg
Copper sulphate	269	92	meq/kg
Cobalt sulphate	516	329	meq/kg
Monoammonium phosphate	815	46	meq/kg
Ferrous oxide	986	549	meq/kg
Finisher minerals and vitamins	5,123	3,357	meq/kg
Weaner minerals and vitamins	6,302	4,292	meq/kg
Dicalcium phosphate	3,813.6 − 10,150	3,098	meq/kg
Sow minerals and vitamins	7,503	5,413	meq/kg
Potassium citrate	7,851	5,703	meq/kg
Mono dicalcium phosphate	1,800 − 5,494		meq/kg
Sodium citrate	8,745	6,334	meq/kg
Defluorinated phosphate	10,436	6,412	meq/kg
Calcium formate	9,000 − 12,069	3,983	meq/kg
Calcium carbonate	19,680 − 20,000		meq/kg
Manganese oxide	10,887	6,678	meq/kg
Sodium bicarbonate	12,870	12,566	meq/kg
Limestone flour	15,044	12,932	meq/kg
Zinc oxide	13,000 − 17,908	16,321	meq/kg
Acids			
Orthophosphoric acid	−7,957	−8,858	meq/kg
Fumaric acid	−6,400 − −4,093	−10,862	meq/kg
Formic acid	−3,473	−13,550	meq/kg
Citric acid	−2,349	−5,605	meq/kg
Ascorbic acid	−4,000 − −2,249	−217	meq/kg
Malic acid	−2,550	−7,214	meq/kg
Lactic acid	−1,498	−5,079	meq/kg
Acetic acid	−141	−2,283	meq/kg
Propionic acid	−5	−1,358	meq/kg
Sorbic acid	120	−220	meq/kg

^*a*^Acid-binding capacity to pH 3.0; ^*b*^Acid-binding capacity to pH 4.0.

Some mineral sources are key contributors to a high ABC value of feed, especially limestone (calcium carbonate), dicalcium phosphate (DCP), mono-dicalcium phosphate (MDCP) and zinc oxide (ZnO). So, it is very important to avoid limestone, DCP, and MDCP in formulating a weaner diet. Lowering calcium levels by decreasing the limestone content of the feed has a huge impact on the buffering capacity of the feed and on improving growth performance (Blavi et al., 2016). Using phytase super-dosing can provide multiple benefits including the reduction of limestone in diets. For example, the calcium and phosphorus levels (for the first 2 wk after weaning) should be reduced to 0.60% to 0.65% and 0.35% to 0.40%, respectively. At the same time, calcium formate (as a Ca source, 9,000 meq/kg) can partially replace limestone (18,000 to 20,000 meq/kg), which will inevitably lead to increased cost but will effectively reduce the feed ABC value.

Dietary supplementation of high levels of ZnO (2,000 to 3,000 mg/kg) has been widely used as an effective approach to reducing the incidences of post-weaning diarrhea (Laskoski et al., 2021). However, using a high dose of ZnO in piglet feeds has been associated with several negative effects including neutralizing the acid in the stomach because of a high ABC, being associated with post-weaning anemia, ZnO toxicity, zinc accumulation in the environment, interacting negatively with phytase, and antibiotic resistance (Maenz et al., 1999; Debski, 2016; Burrough et al., 2019). The maximal of 150 ppm zinc in the feed will be effective in June 2022 and the use of a high dose of zinc will be regulated in Canada. Although it becomes feasible to reduce the effective dosage of ZnO to combat post-weaning diarrhea and at the same time mitigate the negative impacts related to the high dosage of ZnO in feeds with the availability of new cost-effective technologies such as organic minerals, nanotechnology, and microencapsulation (Brown et al., 2019; Wang et al., 2022), there is still a need to develop strategies to replace higher doses of zinc in the feeds.

Organic acids have negative ABC (e.g., citric acid: −4,000 meq/kg and formic acid: −6,400 meq/kg). So, the addition of organic acids can decrease the ABC value of feed and then lower stomach pH (Desai et al., 2007). Sciopioni et al. (1978) reported a reduction in stomach pH from 4.6 to 3.5 with the addition of 1% citric acid and a pH reduction from 4.6 to 4.2 with 0.7% fumaric acid in the diet. However, we should consider the palatability of organic acids. Citric acid and tartaric acids improved feed preference; a high inclusion of some organic acids (e.g., formic acid), however, may reduce feed intake (Suarez et al., 2010). Higher early feed intake is very important for weaned piglets for promoting gut development and supporting better growth performance later on. On the other hand, inorganic acids (e.g., hydrochloric or phosphoric acid) can also reduce stomach pH but may also decrease feed preference (Suarez et al., 2010). Except for inhibition of pathogenic bacteria and reduction of ABC, organic acids can also act as an energy source in the gut of pigs as these are the intermediary products of tricarboxylic acid and improve mineral utilization (Pearlin et al., 2020). Therefore, several factors, including ABC value, palatability, bacteria inhibition, and other physiological functions, should be considered in the selection of organic acids in feeds.

Cereals and cereal by-products have a lower ABC when compared with the sources of minerals and proteins. So, it is important to keep feed crude protein levels as low as possible while maintaining a good, balanced supply of amino acids by using high protein raw materials and synthetic amino acids. When compared with fishmeal and soybean meal, potato protein, wheat gluten, and corn gluten have a lower ABC and therefore are highly recommended in weaner piglet feeds. However, potato protein, wheat gluten, and corn gluten may negatively affect feed intake as those protein sources are less preferred than fishmeal and soybean meal by piglets (Solà-Oriol et al., 2011). Moreover, it is not necessary to reject good high-value raw materials simply based on apparently high ABC as there are other ways to reduce the ABC of the feed.

Selecting ingredients to lower ABC is critical for optimizing the digestive function of the immature piglet. Although there is no clear-cut recommendation on the ABC values for nursery diet, it is critical to look at the total blend of raw materials available, their inclusion levels, costs, feed preference as well as the targeted feed ABC value and then make the final decision based on science and field experience.

## Using Innovative Chemical and Biological Approaches to Detoxify Vomitoxin (DON)

The mycotoxin, deoxynivalenol (DON), commonly occurs on *Fusarium*-infected cereal grains (e.g., corn, wheat, barley), and the incidence of DON contamination of grains has been increasing in recent years (Biomin, 2021). It has been estimated that direct and secondary losses resulting from Fusarium Head Blight (a fungal disease of cereal crops, which also is an indicator of DON contamination) range from $50 to $300 million each year in Canada (Alberta Agriculture and Forestry, 2012). Moreover, the mycotoxin contamination in feeds and feed ingredients can reduce feed intake and compromise the immune system, which can make animals more susceptible to pathogens. Minimizing mycotoxin contamination in feeds is an important component to control post-weaning diarrhea. Typical negative effects of mycotoxin consumption include reduced feed intake, digestive dysfunction (e.g., gastroenteritis, gastrointestinal tract lesions, reduced nutrient absorption), immune suppression, and reduced growth performance (Sergent et al., 2006; Pinton et al., 2008; Johnston et al., 2010; NRC, 2012) with the primary physiological effect dependent on the mycotoxin present. DON levels as low as 0.6 to 2.0 ppm in complete feed cause a reduction in feed intake and growth rate (Pinton et al., 2008; Johnston et al., 2010). In addition to reduced feed intake and growth performance, consuming DON-contaminated feed results in damage to the intestinal tract epithelial cells resulting in alteration of intestinal growth and barrier function as well as increased susceptibility to enteric pathogen challenge (Pinton et al., 2012; Ghareeb et al., 2015). Damage to the intestine also results in a reduction in nutrient absorption (Ghareeb et al., 2015). Once absorbed, DON inhibits protein synthesis, causes kidney and liver damage, and can suppress immune function resulting in decreased ability to resist disease challenges (Chaytor et al., 2011). In general, the negative effects of mycotoxins are greater in younger animals (Chaytor et al., 2011). While strategies have been developed to reduce the effects of some mycotoxins (e.g., aflatoxin), such as toxin binders, these have limited effect on mitigating the negative effects of DON (Beaulieu et al., 2009). There is a need for effective and economical methods to reduce the impact of DON in feed and feed ingredients. Chemical approaches, such as the use of sodium metabisulfite (SMBS) (Rempe et al., 2013), and biological approaches, such as the use of microorganisms for detoxification (Yu et al., 2010; Li et al., 2011), have shown promise in reducing DON.

Sulphite reducing agents, including sodium sulphite (Na_2_SO_3_), sodium bisulphite (NaHSO_3_), and SMBS, have the capacity to cleave disulphide cross-linkages (Truong et al., 2016). Both in vitro and in vivo studies have demonstrated that SMBS is effective in DON detoxiﬁcation (Table 2). Specifically, it has been shown that SMBS can destroy 70% to 100% of DON in processed grains or feeds in vitro with 0.45% to 0.9% levels at pH around 6.5 but not under acidic conditions (Dänicke et al., 2010a, 2010b; Schwartz et al., 2013; Frobose et al., 2015). Frobose et al. (2015) reported that adding a SMBS-based feed additive and pelleting can help overcome some of the negative effects of DON in the nursery pigs fed with naturally contaminated dried distillers grains with solubles (DDGS) (Frobose et al., 2015). Hydrothermally processing DON-contaminated diets with 1.0% SMBS restored ADFI and improved G:F in the nursery pigs (Frobose et al., 2017). Shawk et al. (2019) also reported that in diets with low DON concentrations (<1.5 mg/kg), SMB-based products increased ADG compared with control diets. Pigs fed high DON diets (4.17 mg/kg) had reduced performance compared with pigs fed low DON. Sodium metabisulfite (0.5%) in high DON diets (manufactured with corn containing an average of 4.17 mg/kg DON) provided a benefit in growth performance with ADG and G:F exceeding growth performance in the low DON diet (manufactured with corn containing an average of 2.46 mg/kg DON) (Becker et al., 2022). Mwaniki et al. (2021) reported that a feed additive containing SMB improved growth performance in the nursery piglets fed diets formulated with naturally contaminated corn (formulated with 5.5 mg/kg DON). The results from the above in vivo studies have demonstrated that feeding a supplement with relatively high levels of SMB to weanling pigs is safe and effective to detoxify DON. Although the response is still present even without pelleting in many situations, heat and moisture during the pelleting process seem to enhance the capacity of SMBS to detoxify DON as pelleted feeds were used in the above studies, suggesting SMBS detoxifying DON during the pelleting process not necessary in the gut. Because SMBS may be degraded quickly under aqueous acid conditions such as pig stomach to form sulfur dioxide and subsequently decompose into sodium oxide and sulfur dioxide (Dänicke et al., 2012), then damaging the metabolism of the liver and the functionality of the immune system, eventually leading to a decrease in health or growth performance (Davis et al., 2022). This may explain why more than 0.35% of unprotected SMBS in the diet can show toxic effects on pigs. Moreover, little SMBS will remain intact in the small intestine where an optimal pH environment exists for SMBS to detoxify DON (Yu et al., 2022). Thus, there is a need to deliver intact SMBS to the lower gut such as the small intestine to detoxify DON effectively through innovative delivery methods (Yu et al., 2021, 2022). Encapsulated SMBS with hydrogenated palm oil was stable in the simulated gastric fluid and allowed a progressive release of SMBS in the simulated intestinal fluid. The released SMBS in the simulated intestinal fluid effectively detoxified DON (Yu et al., 2021). However, the efficacy of DON detoxification by microparticles needs to be further investigated with pig experiments. Further, feeding high SMBS in the diet can decrease the bioavailability of thiamin (Til et al., 1972); therefore, thiamin is usually supplemented at greater concentrations or with a protected form in diets that are supplemented with SMBS.

**Table 2.** Summary table of relevant literature on effects of sodium metabisulfite (SMBS) on deoxynivalenol (DON) levels and growth performance of pigs

In vitro or in vivo	Diets/ingredients	Heat and humidity	Mash or pelleted	DON concentrations (ppm or mg/kg)	SMBS concentrations (g/kg or %)	Effects	References
In vivo	Corn-soybean meal	N/A	Phase.1: Pelleted, Phase.2: Meal	Low: 1.12 ppm High: 2.34 ppm High + SMBS: 1.44 ppm	0.5%	Improve feed efficiency, reduce total removals and mortality.	Becker et al., 2022
In vivo	Corn-soybean meal with corn been contaminated	mash	Exp.1: Pelleted, Exp.2: Mash	PC: 0.29 ppm NC: 2.86 ppm NC+SMBS: 1.21 ppm	3 g/kg	Greater G:F and ATTD of dry matter, gross energy, and crude protein.	Mwaniki et al., 2021
In vivo	Barley-corn-soybean	N/A	Mash	Control: 0.29mg/kg Don: 4.49 mg/kg Control+ SMBS: 0.43 mg/kg Don + SMBS: 3.96 mg/kg	3%	Reduce AID of some nutrients and intestinal absorption of DON.	Bouchard et al., 2019
In vivo	Corn-soybean meal	N/A	N/A	<0.5 mg/kg	0.15%, 0.25%, 0.5%	Higher concentration of SMB for a longer duration; Have improved growth performance.	Shawk et al., 2019
In vivo	Wheat-soybean meal	Pelleted at 82 °C with a minimum conditioner retention time of 45 s	Pelleted	PC: <0.5 mg/kg NC: 4 mg/kg NC+SMBS: 0.35 mg/kg	1%	DON was decreased by 92% when pelleted with SMB; Can alleviate DON effects on growth, restore feed intake and improve feed efficiency.	Frobose et al., 2017
In vivo	Corn-soybean meal-DDGS, DDGS is the source of DON	N/A	Mash and Pellet	PC: <0.5 mg/kg NC: 3 mg/kg NC+0.25% SMBS: 3 mg/kg NC+0.5% SMBS: 3 mg/kg	0.25% and 0.5%	No significant pellet × SMB interactions on growth performance and BW, but pelleting improved G:F and ADG, SMBS increased ADG.	Frobose et al., 2015
In vivo	DDGS	N/A	Mash and Pellet	PC: <0.5 mg/kg NC: 4.8 mg/kg NC+ crumbled DDGS: 4.8 mg/kg NC+crumbled DDGS + 0.5% SMBS: 3 mg/kg	2.5% (final concentration in diet: 0.77%)	Including SMB prior to pelleting DON-contaminated DDGS increased (*P* < 0.01) ADG and ADFI.	Frobose et al., 2015
In vivo & vitro	Triticale	18 °C, 15% moisture for 4 wk	N/A	0.156, 2.312, 0.084 and 0.275 mg/kg	5%	Wet preservation in the presence of SBS could reduce DON level; restored growth performance to the level of their counterparts.	Dänicke et al., 2010a
In vitro	DDGS	Autoclave 1h at 121 °C	N/A	20.6 mg/kg	0.5%, 1%, 2.5%, 5%, 5% with 100 mL/kg distilled water	9.8% reduction in DON, 82% were achieved when 5% SMBS were added before autoclaving.	Frobose et al., 2015
In vitro	DDGS	Pelleting at 66 °C and 82 °C with retention times of 30 s and 60 s within temperature	N/A	20.5 mg/kg	1%, 2.5%, and 5%	Pelleting with SMBS inclusion can reduce DON level.	Frobose et al., 2015
In vitro	Wheat kernels	18 °C, 15%, 17.5% and 20% humidity		2.09 mg/kg dry matter	5%	Reduce 1.2–4.3% initial DON concentration, higher SBS-to-DON ratio and higher moisture content favour the derivatization of DON to DONS.	Dänicke et al., 2010b
In vitro	Triticale	13% and 15% moisture and stored for up to 63 d		6.63 mg/kg	1%, 2%, 3%, 4%, 5%	DON concentration decreased with increasing amounts of supplemented SBS, moisture and longer duration.	Dänicke et al., 2009
In vitro	Wheat	100 °C, 22% moisture content for 15 min		7.6 mg/kg	1%	Reduce the DON-concentration to 0.28 mg/kg.	Dänicke et al., 2005

Biological approaches, such as using microorganisms to convert the toxins to non- or less toxic compounds, have become an attractive choice recently due to their high specificity, efficacy, and environmental soundness (Awad et al., 2010; He et al., 2016; Pierron et al., 2016; Vanhoutte et al., 2016; Zhu et al., 2016; Tian et al., 2022). It has been shown that the higher tolerance for DON observed in ruminants is due, in part, to the conversion of DON to nontoxic metabolites by rumen microorganisms (Chaytor et al., 2011). For instance, a bacterial strain BBSH797 from the bovine rumen (Fuchs et al., 2002) could transfer DON into its metabolite DOM-1. Additionally, some bacteria from the poultry industry also have the potential to detoxify DON into DOM-1, including a Clostridium sp. WJ06 from goose intestine in China (Li et al., 2017), a Bacillus sp. LS100 from the chicken intestine (Yu et al., 2010), and an Eggerthella sp. DII-9 from the chicken intestine (Gao et al., 2018). In general, the high pre-gastric bacterial count in both ruminants and poultry may be a major factor with respect to DON tolerance in these species (Maresca, 2013). Their detoxification principle is that the C12-C13 epoxy group is the main toxicity site of DON (Karlovsky, 2011), and DON can be deepoxidized to the metabolite DOM-1, which is considered to be a detoxification product. Indeed, many species of bacteria have been shown to possess the capability to enzymatically degrade mycotoxins (Shetty and Jespersen, 2006). The described animal gut microbes deeply oxidize DON under anaerobic conditions, which limits its practical application and only a few of them were identified that could detoxify DON-contaminated diets in vivo. A bacterial strain Coriobacteriaceum DSM 11798 (the active ingredient in Biomin BBSH 797) can be used as a feed additive in diets to remove the toxic effects of DON-contaminated diets in pigs by detoxifying DON to DOM-1 (Sayyari et al., 2018). Previously isolated microorganisms, including Bacillus sp. LS100, has been shown to have DON detoxifying properties in vitro (Yu et al., 2010). The concept of using the isolate for in vivo detoxifying DON has also been proven (Li et al., 2011) and found that microbial detoxification of contaminated feed could eliminate DON’s toxic effects on pigs. A U.S. patent has been granted for utilizing the bacterial isolates (Zhou et al., 2014). Since isolate LS100 possesses high efficiency and stability in detoxifying DON, direct feeding of the isolate through feed provides a unique opportunity for developing an effective microbial agent for field application to detoxify DON, which requires further pig studies to confirm the efficacy of detoxification in the pig gut.

## Conclusion

Weaned piglets face many stressors including bacterial pathogens, oxidative stress, and inflammation. The withdrawal of antibiotics from feeds can result in challenges to control post-weaning diarrhea and an integrated approach should be taken to control post-weaning diarrhea. Regarding antibiotic alternatives, using lower doses of essential oils (e.g., combination with other bioactive compounds and microencapsulation) could prevent inflammation and “leaky gut” induced by lipopolysaccharide instead of higher doses required for their killing pathogens properties. With elevated pH levels in the stomach of weaned piglets, it is critical to look at the total blend of raw materials available, their inclusion levels, costs, feed preference as well as the targeted feed ABC value. Minimizing mycotoxin contamination in feeds is an important component to control post-weaning diarrhea. Chemical approaches, such, as the use of sodium metabisulfite and biological approaches, such as the use of microorganisms for detoxification, have shown promise in reducing vomitoxin. These strategies reviewed in this manuscript may be considered important parts of an integrated approach to control post-weaning diarrhea in piglets.

## About the Authors

**Liuqin He**, Professor of College of Life Sciences, Hunan Normal University. She received her Ph.D. degree in ecology from the University of Chinese Academy of Sciences in 2018. She won Young Elite Scientists Sponsorship Program by China Association for Science and Technology, “Huxiang Young Talents Plan” of Hunan Province, and the Natural Science Foundation for Outstanding Youth Scholars of Hunan Province. She mainly engages in amino acid nutrition regulation and animal health. E-mail:285687180@qq.com

**Xiaoya Zhao** obtained her Ph.D. degree at the Department of Animal Science at the University of Manitoba in 2022 and she is currently working at South China Agricultural University as a postdoctoral fellow. During her Ph.D. study, her primary research interests are located in the roles of gut chemosensing in mammals’ growth and health, and she is also interested in identifying antibiotic alternatives. For her postdoctoral research, her interest turned to oncolytic virus therapy in cancer in pets. Currently, her postdoctoral research is about the impact of the tumor microenvironment on oncolytic viral therapy.

**Li Jianzhong** obtained his Ph.D. degree in aquatic biology from Sun Yat-Sen University in June 2003 and worked as a postdoctoral researcher at the National Institute of Basic Biology in Japan from October 2006 to October 2008. He is working at the School of Life Sciences, Hunan Normal University as a professor and a doctoral supervisor. His research interests are animal nutrition and gut health. He has published about 50 peer-reviewed journal articles as the first author or corresponding author and participated in the application of 6 national patents in China.

**Dr. Chengbo Yang** is n Associate Professor in Livestock Nutrition and Nutritional Biochemistry in the Department of Animal Science, University of Manitoba. Dr. Yang obtained his Ph.D. in monogastric animal nutrition at the University of Guelph in 2011. He joined the University of Manitoba in 2016 after several years of working in the industry. He is conducting research in the area of gut health and nutrient utilization relevant to nonruminants. He has acquired about 10.0 million dollars in research funding as PI and co-PI from NSERC, Canada Foundation for Innovation, USDA, Swine Cluster III, and other agencies. He currently serves as an editorial board member for two journals including the Canadian Journal of Animal Science. He has provided training to over 50 HQPs including high school students, undergraduate, and graduate students, postdoctoral fellows, technicians, and research associates, and published 89 peer-reviewed scientific publications. Dr. Yang received with 2018 Canadian Society of Animal Science Young Scientist Award, the 2018 Terry G. Falconer Memorial Rh Institute Foundation Emerging Researcher Awards for Natural Sciences, and the 2019 Merit Award in Research, Scholarly Work, and Creative Activities at the University of Manitoba.
